# Health surveillance assistants as intermediates between the community and health sector in Malawi: exploring how relationships influence performance

**DOI:** 10.1186/s12913-016-1402-x

**Published:** 2016-05-03

**Authors:** Maryse C. Kok, Ireen Namakhoma, Lot Nyirenda, Kingsley Chikaphupha, Jacqueline E. W. Broerse, Marjolein Dieleman, Miriam Taegtmeyer, Sally Theobald

**Affiliations:** Royal Tropical Institute, P.O. Box 95001, 1090 HA Amsterdam, The Netherlands; VU University Amsterdam, Athena Institute for Research on Innovation and Communication in Health and Life Sciences, De Boelelaan 1081, 1081 HV Amsterdam, The Netherlands; Research for Equity and Community Health (REACH)Trust, P.O. Box 1597, Lilongwe, Malawi; University of Livingstonia, C/o CCAP Synod of Livingstonia, P.O. Box 112, Mzuzu, Malawi Africa; Liverpool School of Tropical Medicine, Department of International Public Health, Pembroke Place, Liverpool, L3 5QA UK

**Keywords:** Health surveillance assistants, Community health workers, Malawi, Performance, Trust, Relationships

## Abstract

**Background:**

There is increasing global interest in how best to support the role of community health workers (CHWs) in building bridges between communities and the health sector. CHWs’ intermediary position means that interpersonal relationships are an important factor shaping CHW performance. This study aimed to obtain in-depth insight into the facilitators of and barriers to interpersonal relationships between health surveillance assistants (HSAs) and actors in the community and health sector in hard-to-reach settings in two districts in Malawi, in order to inform policy and practice on optimizing HSA performance.

**Methods:**

The study followed a qualitative design. Forty-four semi-structured interviews and 16 focus group discussions were conducted with HSAs, different community members and managers in Mchinji and Salima districts. Data were recorded, transcribed, translated, coded and thematically analysed.

**Results:**

HSAs had relatively strong interpersonal relationships with traditional leaders and volunteers, who were generally supportive of their work. From the health sector side, HSAs linked to health professionals and managers, but found them less supportive. Accountability structures at the community level were not well-established and those within the health sector were executed irregularly. Mistrust from the community, volunteers or HSAs regarding incentives and expectations that could not be met by “higher levels” undermined support structures and led to demotivation and hampered performance. Supervision and training were sometimes a source of mistrust and demotivation for HSAs, because of the perceived disinterest of supervisors, uncoordinated supervision and favouritism in selection of training participants. Rural HSAs were seen as more disadvantaged than HSAs in urban areas.

**Conclusions:**

HSAs’ intermediary position necessitates trusting relationships between them and all actors within the community and the health sector. There is a need to improve support and accountability structures that facilitate communication and dialogue, increase trust and manage expectations and thereby improve interpersonal relationships between HSAs and actors in the community and health sector. This would maximize the value of HSAs’ unique intermediary position and support them to deliver equitable health services. This is particularly important in rural areas, where HSAs often constitute the only point of contact with health services, yet report limited support from the health system.

**Electronic supplementary material:**

The online version of this article (doi:10.1186/s12913-016-1402-x) contains supplementary material, which is available to authorized users.

## Background

In many countries, community health workers (CHWs) are a key component of strategies to achieve universal health coverage, through extension of primary health services to underserved communities at low costs in contexts of chronic financial and human resource shortages [1].

In Malawi, a large number of different types of CHWs link communities with the health sector [2]. The largest group is the government paid cadre of health surveillance assistants (HSAs), comprising 30 % of the health workforce [3] and totalling 9,137 [4]. HSAs are recruited by the government, must have secondary school level education and receive 12 weeks training [2, 5]. Once employed, they are supposed to reside in their catchment area, working mainly in health promotion and prevention for a population of about 1,000 [6]. From 2008, HSAs’ curative tasks have been expanded. HSAs working in hard-to-reach areas^1^ conduct integrated community case management of childhood illnesses (iCCM) [5, 7, 8]. HSAs are supervised by senior HSAs or (assistant) environmental health officers [7]. They are attached to a hospital or health centre, but are supposed to spend most of their time in the community. HSAs are supported by village health committees (VHCs), consisting of ten unpaid village representatives elected by the community, and other volunteers, such as members of HIV support groups and traditional birth attendants [2, 9].

Evidence from various countries shows that CHWs can effectively deliver key health interventions [10]. Although large scale studies on effectiveness of HSAs in Malawi are missing, evidence on HSAs’ positive effect on immunization rates [11] and access to anti-retroviral treatment for HIV [12] is available. Given the ongoing human resources shortage in Malawi [13, 14], HSAs will remain an essential cadre in driving forward efforts to achieve universal health coverage and it is important to better understand which factors influence their performance.

Earlier studies identified several constraints to HSAs’ motivation and job satisfaction, which were negatively influencing performance. Factors related to the health sector, such as lack of supplies and infrastructure [5–7, 9, 11, 15, 16], unclear or too many roles and responsibilities [3, 9, 15, 16] and inadequate human resource management related to training, supervision, incentives and career development [3, 5, 7, 9, 11, 15, 16] have all been identified. Factors related to the community were (less often) identified: inadequate support from community volunteers [9] and unrealistic expectations from the community regarding HSAs’ roles could hamper HSAs’ motivation and job satisfaction [15].

CHWs have a unique intermediary position in between their clients and the broader community (further referred to as the community) and health professionals at the facility level, including their supervisors (further referred to as the health sector). Therefore, CHWs are often seen as the most strategically placed cadre to increase equitable access to health care [17, 18]. However, this intermediary position may have disadvantages for CHWs, for example when the expectations of the community and the health sector differ with respect to the role of HSAs, leading to high workload or demotivation [19]. There has been growing interest in how CHWs’ intermediary position shapes performance [20, 21] as their positionality necessitates good interpersonal relationships, defined as interactions between two individuals [22, 23], with community members as well as practitioners within the health sector. Relationships within the community enable HSAs to optimally engage with different community actors, promoting healthy behaviour [18, 24–26]. Links to and being part of the health sector enable HSAs to optimally serve their communities by providing referral, supervision and supplies, and enhancing their credibility [27–30]. Health systems comprise a complex web of relationships whose overall functioning is influenced by the institutions, particularly trust, that govern human behaviour [22]. We define trust as “the optimistic acceptance of a vulnerable situation in which the trustor believes the trustee will care for the trustor’s interest” [31]. Trust could be built by personal behaviours and organizational practices that provide space for engagement and open dialogue [32]. Factors that have been found to influence trust of health workers are perceived organizational support, communication, procedural justice and feedback from upper levels [33, 34]. The recognition that health workers are social actors points to the importance of trusting relationships, defined as respectful, fair and cooperative interactions between individuals [22, 23]. Thus, understanding the factors that influence HSAs’ trusting relationships with different actors in the community and health sector is important in order to analyse and improve HSA performance.

In Malawi, earlier research revealed constraints regarding HSAs’ interpersonal relationships with actors in the health sector related to inadequate supervision and communication, leading to demotivation [9, 11, 35] and mistrust because of problems in drug supply [7] and inadequate support mechanisms for HSAs conducting iCCM in hard-to-reach areas [15]. Improved communication between HSAs and the health sector via mobile phones was reported to increase self-confidence of HSAs and community trust [36].

Thus far, an in-depth assessment of factors influencing interpersonal relationships and the implications for HSA performance in hard-to-reach areas is lacking. This qualitative study aims to obtain in-depth insight into the facilitators of and barriers to interpersonal relationships between HSAs and actors in the community and health sector in hard-to-reach settings in two districts in Malawi, in order to inform policy and practice on optimizing HSA performance.

## Methods

Focus group discussions (FGDs) and semi-structured in-depth interviews were conducted from July till September 2013 in Mchinji and Salima districts of Malawi. Both districts are situated in the central region and have urban and rural areas. The sample of participants was drawn from two traditional authorities^2^ in Mchinji and three in Salima, all defined as hard-to-reach [2]. Study respondents were purposefully sampled to represent respondents from the side of the community (women with under-five children, volunteers, traditional birth attendants and traditional leaders), health sector (district managers, health centre in charges and representatives of non-governmental organizations (NGOs)) and HSAs themselves, including senior HSAs, and to ensure diversity in gender, age and job experience. Respondents were identified with the help of district level staff. A total of 16 FGDs and 44 interviews were conducted (Table 1).

**Table 1:** Overview of focus group discussions and interviews

Method	Participants	Total No. of respondents (Total no. of FGDs)
Focus Group Discussions	HSAs	19 (3)
	Women with under five children	70 (7)
	Volunteers	48 (6)
Total		137 (16)
Semi-structured in-depth interviews	HSAs	5
	Senior HSAs	3
	Mothers	1
	Traditional birth attendants	6
	District level managers and health staff	13
	Health centre in charges	2
	NGO representatives	9
	Traditional leaders	3
	Volunteers	2
Total		44

Semi-structured topic guides were developed in English, translated into Chichewa and back-translated for consistency. Topic guides were piloted in an area not included in the study and minor adaptations to questions and probes were made. FGDs and interviews included questions on demographic information; tasks; career; relationships with the community and health sector; training; supervision; monitoring and evaluation and referral. The questions focused on factors related to the design of the HSA programme that influenced HSA performance. An example of a topic guide (for interviews with HSAs) is provided in Additional file 1, others are available upon request. The performance of HSAs was defined at two levels: HSA level (this included self-esteem, motivation, attitudes, competencies, guideline adherence, job satisfaction and capacity to facilitate community agency as characteristics of performance) and end-user level (this included utilization of services, health seeking behaviour, adoption of practices promoting health and community empowerment as characteristics of performance) [19]. Data were collected by a trained research team familiar with conducting qualitative research in rural Malawian contexts. Study participants gave informed oral or written consent. Daily debriefing sessions with data collectors were held to discuss key findings, refine lines of inquiry and summarize field notes and observations. FGDs and interviews were digitally recorded, transcribed and translated into English. A sample of transcripts was randomly checked against recordings.

Initially ten transcripts were read in pairs to identify key themes. Proposed key themes were discussed and agreed upon within a team of four researchers and a coding framework was developed. For the purpose of this article, we only focused on factors that influenced personal relationships and related this, where possible, to the characteristics of HSA performance as presented above, with a focus on HSA related characteristics such as motivation and satisfaction (within the context of the broader framework on all possible factors influencing HSA performance [19]). The analytical process included inductive thematic analysis and open coding [37]. Transcripts were coded using Nvivo (v.10) software and emerging themes were discussed and coding refined. The coded transcripts were further analysed and summarized in narratives for each theme. Themes were categorized into factors influencing HSAs’ relationships with communities, factors influencing HSAs’ relationships with the health sector and cross-cutting factors influencing relationships. Study findings were discussed and validated with the two district health offices through feedback meetings.

The study was approved by the Royal Tropical Institute Ethical Review Committee in the Netherlands (S45B) and the National Health Sciences Research Committee in Malawi (NHSRC #1168).

## Results

Interpersonal relationships between HSAs, the community and health sector were found to be influenced by several factors. First, programme design elements influencing HSAs’ relationships with the community are presented, followed by those influencing HSAs’ relationships with the health sector. Cross-cutting factors, categorized as trust, communication and dialogue, and expectations (as summarized in Table 2), are presented throughout. Where possible, the link between relationships and HSA performance was made. Illustrative quotes are used to depict the main themes.

**Table 2:** Programme design and cross-cutting factors influencing HSAs’ relationships with the community and health sector

Programme design elements influencing relationships	Cross-cutting factors influencing relationships		
	Trust	Communication and dialogue	Expectations
HSAs’ relationships with the community			
Nature of HSAs’ position and role	Honesty, familiarity, good attitudes, reliability, respect and time spent in the community enhanced community trust, and if not present, hampered community trust in HSAs	When HSAs were either from or resided in the communities, this supported opportunities for ongoing communication and dialogue Increasing amount of facility-based tasks or prioritization of agricultural work undermined communication and dialogue between HSAs and communities	
Support from the community	Support from traditional leaders enhanced HSAs’ credibility, which enhanced community trust in HSAs Mistrust from volunteers towards HSAs about financial incentives hampered community trust in HSAs	Support from traditional leaders facilitated communication and dialogue between HSAs and community members, for example during community meetings	Volunteer support helped HSAs in managing community expectations, improving HSAs’ relationships with the community Expectations of volunteers that could not be met, regarding financial and other incentives, training and supplies, hampered HSAs’ relationships with the community and health sector
Community monitoring and accountability structures	Within some programmes, e.g. iCCM, a formal system was in place to support and monitor drug distribution through the VHC, in others this was absent or mediated by traditional leaders. This study revealed no further information on underlying factors influencing HSAs’ relationships with the community.		
HSAs’ relationships with the health sector			
Support from other health workers, managers and NGOs	Disrespect from other health workers led to HSA and community mistrust towards the health sector Support from other health workers enhanced credibility and community trust towards HSAs Perceived lack of management support and favouritism regarding supplies led to mistrust from HSAs towards management	Disrespect from other health workers hindered communication between other health workers and HSAs	HSAs’ expectations with respect to supplies, bicycles, and housing issues were not met (particularly in rural areas)
Training	Perceived favouritism regarding training led to mistrust from HSAs towards management		HSAs’ training expectations were not met – particularly in rural and hard to reach areas
Supervision	Lack of care and insight of supervisors into HSAs’ situation led to mistrust of HSAs towards supervisors	Supervision with a negative approach and without feedback hindered communication between HSAs and supervisors/management	
Referral		Lack of feedback after referral hindered communication between HSAs and the health sector	
Monitoring and accountability structures	Monitoring and accountability structures from the side of the health sector were programme specific and irregularly conducted because of resource constraints. The study revealed no further information on underlying factors influencing HSAs’ relationships with the health sector.		

### HSAs’ relationships with the community

Many HSAs acknowledged the importance of having good interpersonal relationships with the community. HSAs reported that time spent in the community, good attitudes and reliability of HSAs positively influenced community’s confidence and trust in them.

*“When there is a good relationship between you and the community, things go well. A good relationship will make service provision better, people respond to you and adhere to whatever you tell them, that’s the vital key. Health service providers must show examples by how they live… You must show respect, be compassionate, and friendly… They* [HSAs] *should be honest, they should be able to stick to and fulfil their promises; otherwise, people tend to lose their confidence in you… and that is bad for service delivery…”* (Interview, HSA, Mchinji)

HSAs and managers reported that the nature of HSAs’ position and role in the community assured a “natural link” between HSAs and the community, which facilitated good relationships. HSAs’ relationships with the community were furthermore facilitated by support systems from the community and to a lesser extent by community monitoring and accountability structures. Within those systems and structures, trust, communication and dialogue, and expectations emerged as cross-cutting factors influencing relationships (Table 2).

### The nature of HSAs’ position and role

Although many HSAs were not staying in their catchment area (as a result of recruitment of HSAs not based on residence in the area of service or lack of appropriate housing), being known and coming from the catchment area was found to be important for enhancing confidentiality, trust and fostering relationships with the community. For example:

*“Whenever a person who is a stranger to the community is conducting a meeting, there is negative feedback; since they are not familiar with the person and what he is explaining… They* [the community] *will disregard whatever the new person says and do not have trust in him. But I believe if they choose the person from the same community the people will have trust in him, believing they* [the HSAs] *will be confidential in service provision and are free with each other because of the longstanding relationship that is there.”* (Interview, HSA, Mchinji)

However, some respondents reported that not all HSAs had a strong bond with their community. This was due to the increasing amount of facility-based tasks or, according to community members, neglect of HSAs’ tasks due to spending a lot of time on agriculture or business.

### Support from the community

#### Support from traditional leaders

Traditional leaders supported HSAs by conducting community-based meetings and disseminating health education messages. This facilitated HSAs’ relationships with the community through enhanced credibility, positively influencing community trust. Traditional leaders reported sanctioning people for non-healthy behaviour, but it was not clear whether this facilitated or hindered HSA performance.

*“As a group village headman, I call for a village development committee meeting, where all village headmen in my community are called to participate. During this meeting, I tell them to sensitize people in their villages on safe motherhood… And those women who deliver on their way to the hospital they are to pay a goat as a punishment for not going to the hospital in time.”* (Interview, traditional leader, Mchinji)

Not all HSAs received support from traditional leaders. Some HSAs reported a lack of support as a result of a lack of incentives for traditional leaders, which constrained HSAs’ relationships with the community and hindered their performance on community mobilization.

#### Support from volunteers

Community-based volunteers, who were members of a wide range of committees (such as VHCs or growth monitoring committees), and often attached to vertical NGO-led programmes, supported HSAs in conducting their daily tasks and reporting on problems that needed HSAs’ attention. Establishing good relationships with traditional leaders and volunteers was reported as a precondition for success of programmes. This emerged as an important theme in the analysis, as clearly articulated by one HSA:

*“They* [programme managers] *should first go through the community leaders of the area they want to implement their project in, who will in turn inform their subjects of the proposed project; then they* [the community leaders] *will identify the volunteers in the community, as the community will have complete trust in the volunteers they have chosen, and when the programme is finally introduced you [HSA] coordinate with the volunteers and you become their supervisors, and it becomes very easy to relate with the community. If you decide to do it alone, you will face a lot of challenges…”* (Interview, HSA, Mchinji)

Volunteers also supported HSAs in dealing with expectations of the community that could not be met. This was mainly related to iCCM, where volunteers assisted HSAs by explaining to the community that adults are not covered to receive drugs from village clinics under the iCCM programme and the reasons behind frequent drug stock-outs in the village clinics. Many respondents acknowledged the importance of support from volunteers in facilitating trusting relationships between HSAs and the community. However, several factors were reported to hinder volunteer support. A lack of incentives for volunteers, including training, was reported to result in attrition and thereby hindering HSAs’ performance. Various respondents pointed to mistrust between volunteers and HSAs when it comes to financial incentives.

*“The benefit which was supposed to go to the volunteers is shared between the HSA and the person who has been engaged to do the work, like giving vaccines to the children; since the person had been engaged like a part-time worker, instead of giving the work and the benefits to the volunteers.”* (FGD, volunteers, Mchinji)

Most volunteers expected some financial remuneration. According to some HSAs and managers, this situation was created by various NGO-led vertical programmes with non-harmonized allowance policies, resulting in confusion on what to expect when volunteering for a certain programme. Expectations of volunteers regarding financial incentives were reported to weaken trusting relationships between HSAs and the community.

“*For our job to be successful, we need to work hand-in-hand with volunteers and yet they do not give them allowances when we take them to outreach to help us and this makes them lose trust in us.”* (FGD, HSAs, Mchinji)

To optimally benefit from volunteers’ support, the same persons were chosen as volunteers for different organizations by the HSA or following advice from the HSA.

*“… So nowadays there are a lot of activities which need volunteers… Organization X, Organization Y, Ministry of Health will need theirs. The volunteers are the same people but we just change names* [titles of volunteers] *… Because if you change the volunteers then the whole village will end up being volunteers… We choose a volunteer who is very active at the community level. So when Organization X comes you choose the same person, for Organization Y you choose the same person. Because you know that this person does not let you down…”* (FGD, HSAs, Salima)

#### Support from the wider community

Generally, community members valued the work of the HSAs. Some managers gave examples of communities assisting in building houses for HSAs, as a result of good interpersonal relationships between the HSA and the community. Some HSAs reported that they failed to serve the community because of lack of supplies and in order to cope with community expectations that could not be met, they preferred to stay outside their catchment areas.

*“… When a child is sick they run to you expecting medicine and if you don’t help then it becomes a problem… As a result we just choose not to live there; we travel. But if they can train us in all things then we can be reliable and the work can be easier.”* (FGD, HSAs, Salima)

### Community monitoring and accountability structures

As indicated above, various voluntary committees had a role in supporting HSAs, but monitoring and accountability was not widely reported as core tasks of these committees. For the iCCM programme, a system was in place, illustrated by the following quote:

*“… To get bulks of drugs, the HSA does not come alone, as he or she is supposed to come with one person from the village committee. These people are chosen by the villagers… The drug box has two keys, one is kept by a member of the community and the other stays with the HSA him or herself…”* (Interview, district manager, Mchinji)

On other issues, more informal strategies, for example linking with traditional leaders, were used to get feedback on health services offered by HSAs. Respondents made no reference to possible influence of monitoring and accountability systems on relationships and HSA performance.

### HSAs’ relationships with the health sector

Some HSAs and community members stressed the importance of the intermediary position of the HSA. Other HSAs preferred to see their position as officially attached to the health sector, and found volunteers, instead of themselves, to be positioned in between the community and the health sector.

*“We are like the messenger between health workers and people in the area connecting them on the problems they face concerning health.”* (Interview, HSA, Mchinji)

*“… The volunteers are like a bridge between villagers and us people who work for the government. Actually to us people who work for the government, we don’t belong there in the villages, we are like visitors, so every problem we encounter whilst we are right there in the village, it is the volunteer who is going to help us.”* (FGD, HSAs, Mchinji)

HSAs’ relationships with the health sector were mainly facilitated through support from other health workers and management or NGOs; and through training, supervision, the referral system and monitoring and accountability structures. Within those programme design elements, communication and dialogue, expectations and trust emerged as cross-cutting factors influencing relationships (Table 2).

### Support from the health sector

#### Support from other health workers

Community members viewed support of other health workers towards HSAs as important for quality assurance purposes. HSAs reported that other health workers both facilitated and hindered their performance. HSAs gave examples of health workers who supported them in conducting outreach clinics. Some HSAs reported that clients were not being treated properly or endured long waiting times before being assisted in health centres, which could possibly lead to mistrust and demotivation of HSAs when they had to refer. Some HSAs reported that disrespect from other health workers led to lack of trust from HSAs towards other health workers, but also from the community towards HSAs, which led to demotivation of HSAs.

*“We are not respected as health workers compared to our friends who also do the same work, like nurses. They* [nurses] *think because our work is based in the villages we are not important… This does not encourage us and communication between us and them* [nurses] *does not work well because they underrate us…”* (FGD, HSAs, Mchinji)

#### Support from management or non-governmental organizations

HSAs in hard-to-reach areas complained about receiving less favourable responses from the management when it comes to supplies, bicycles, getting selected for training and housing issues as compared to HSAs living in urban areas.

*“We HSAs in the hard-to-reach areas are also not considered for trainings; whenever there are trainings, they only take the HSAs at the district; in that case they are better oriented than we who are in the fields.”* (Interview, HSA, Mchinji)

Support of NGOs was more positively evaluated by HSAs, although in the case where HSAs felt bypassed by NGOs, they reported that they could sabotage the NGO’s programmes.

### Training

Continuous training could facilitate HSAs’ relationships with other health workers or enhance respect from other health workers because of upgraded knowledge of HSAs. However, on the job training was often not conducted. Specific training courses, often provided by NGOs, were available, but were sometimes a source of demotivation or dissatisfaction for HSAs, as a result of perceived favouritism in selection of training participants or trainings considered to be too short to capture everything. As a result, HSAs reported that some colleagues deliberately underperformed.

*“… When you know something like family planning methods without trainings, HSAs tend not to work because they did not go to the trainings and did not get the allowances their friends got at the trainings. So, one may not be willing to work because they did not get that money.”* (FGD, HSAs, Salima)

### Supervision

Although most HSAs reported that supervision was in place, it was often irregularly implemented. Supervision was often reported to be negative and lack feedback, leading to poor relationships and HSA demotivation and dissatisfaction:

*“If you have done well, they should tell you that you have done well and if you did not do well they should also tell you that you did not do well. That is motivating, and they should not just be telling you on areas which you have not done well, that is not good.”* (FGD, HSAs, Mchinji)

Lack of supervision was reported to promote absenteeism and decrease motivation. HSAs’ wish to be supervised and their plea for supervisors to better understand the circumstances in which they operated was an emerging theme. Some HSAs were mistrusting their supervisors, feeling disconnected because of the different worlds supervisors and HSAs came from, lived in, and had to cope with.

*“… Some bosses were born and raised up in town and when the terrain is bad they just send representatives. They would only come when people from headquarters are visiting so that they would smoothen things up before the visitors come… So sometimes we wonder how people who travel by car would complain about the distance. If they are complaining, what about us who have to cycle to get vaccines? Sometimes we feel we are not being treated as Malawians.”* (Interview, HSA, Mchinji)

### Referral

Generally, there was a referral system in place: some areas had paper referral forms. Feedback to the HSA post referral was not common practice, hindering communication between HSAs and the health sector.

*“When a person has been referred and has received the treatment and is discharged, they don’t write on the discharge sheet the medication they gave them for our records, so it becomes very difficult to do the follow ups, we would like if they do this as at the moment there is breakdown in communication.”* (Interview, HSA, Mchinji)

### Monitoring and accountability

With regard to monitoring and evaluation, the reporting system was clearly described by various respondents. Reporting was conducted separately for different programmes, increasing workload, although this was not referred to as a hindering factor for relationships or HSA performance. Review meetings were conducted, but frequency depended upon budget availability.

*“When we have funds available, we have quarterly meetings where we invite the traditional authorities for the whole district, two HSAs from every health facility and discuss what has happened in the communities in the last quarter, and then people discuss the successes, the challenges and the way forward.”* (Interview, district manager, Mchinji)

## Discussion

**Fig. 1:**
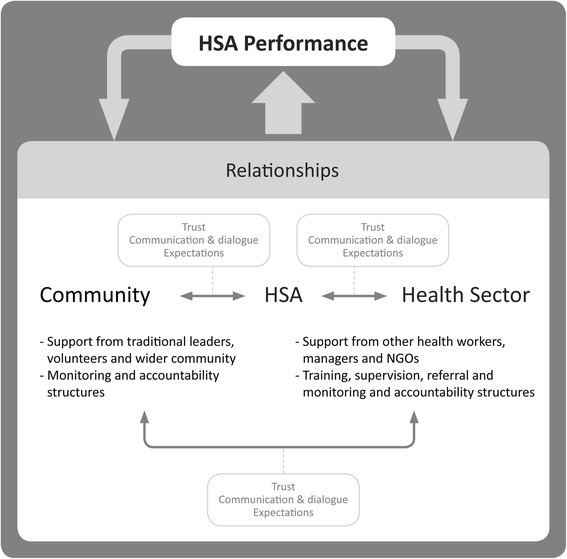
Overview of relationships and their underlying factors, affecting HSA performance

Our study highlights the range of different actors and programme design elements shaping HSAs’ relationships with both the community and health sector which in turn influence HSA performance. As shown in Table 2 and Fig. 1, trust, communication and dialogue, and expectations (of managers, supervisors, HSAs, volunteers and communities) were key cross-cutting factors influencing relationships and shaping performance, in line with findings of other studies focusing on public sector or health workers [23, 33, 34, 38]. From the community side, traditional leaders and volunteers could play a major supporting role for HSAs, with differences across contexts depending on the strength of relationships and availability of support structures (including incentives). From the health sector side, support from other health workers, managers and NGOs and training, supervision, the referral system and to a lesser extent monitoring and accountability structures were identified as important programme design elements facilitating relationships. However, many of these systems and structures seemed to be undermined by a lack of trust, unmet or unrealistic expectations and poor communication and dialogue, leading to poor interpersonal relationships and negatively influencing HSA performance. The perceived performance of HSAs also influences the levels of trust of the community or health sector. Trust in HSAs is also inextricably linked with the trust that the community has in the general health system, this has not been covered by our study (Fig. 1).

### Trust

Trust is an important factor affecting interpersonal relationships. From the community side, trust can be enhanced by shared values between community and health workers and health workers’ attitudes and competencies [22]. Our study found that contributors of mistrust, such as HSAs not being embedded in their communities, undermined shared values and supportive relationships. The distribution of (financial) incentives led to mistrust^3^: from volunteers towards HSAs and from HSAs towards their supervisors and district level staff. Actors within the health system thought that others misused allowances meant for them. This stems from the situation that allowances for travel and training are widespread and seen as income-supplement for health staff in Malawi [39]. With respect to allowances, another study in Malawi concluded that programmes working with volunteers with no or limited livelihoods bring financial burden to volunteers and their households; supporting the argument about the importance of financial incentives for this group [40].

Supervision and training could facilitate trusting relationships between HSAs and the health sector, but were a source of mistrust for some HSAs, and in some cases could even lead to sabotage. In these extreme cases, HSA and the broader programme performance could seriously suffer. Our study did not include HSAs’ perspectives on the trainings they attended. It would be interesting to learn which training contents (such as communication skills, views on professionalism) and approaches could enhance HSAs’ relationships with both the community and health sector. Recognition from supervisors and managers of HSAs’ difficult work in hard-to-reach areas was an important motivator for HSAs and brought credibility, as also found in other settings [18]. HSAs working in hard-to-reach areas felt disfavoured when compared to their urban counterparts with respect to training and other incentives; and there is need for further investigation to assess the extent of this and whether a negative equity scenario is at play (i.e. rural HSAs have less support and work in a more challenging scenario where they are expected to reach remote and marginalized communities). Thus, rural HSAs may face particular challenges in realizing the strengths of their intermediary position, and need additional support.

Every trusting relationship sets up a potential power relation between those involved, which may cause conflicts, or even exploitation or corruption and may initiate a vicious cycle of mistrust [22]. HSAs’ intermediary position means that they are at risk of being linked to cycles of mistrust, which was confirmed by our study.

### Communication and dialogue

Trust is intertwined with communication and dialogue. Within relationships, trust develops from social interactions and ongoing communication and dialogue. A study on CHWs in India reported that open communication with supervisors was a critical element of building trusting relationships and enhancing motivation [41]. Our study found that communication and dialogue, sometimes facilitated by traditional leaders, supported relationships between HSAs and the community. However, poor communication and dialogue between HSAs and their supervisors hindered relationships of HSAs with the “upper level”. Our study revealed limited high-trust management practices, such as participation, problem-solving, feedback and open communication [22]. This led to low workplace trust [42]; in this case HSAs’ low levels of trust in the health sector (the employer and supervisor) hampered motivation and performance. Other studies have also demonstrated that low workplace trust can have a negative influence on trusting relationships between health workers and their clients [32, 42].

### Expectations

The new curative tasks that HSAs conducted in the field of iCCM sometimes resulted in community expectations that could not be met by the HSA. This led to demotivation and dissatisfaction, and in certain cases reluctance to stay in the community, which in turn hinders HSAs’ embedment in the community and their ability to reach out to groups with limited access to health care [9]. More research is needed to assess if community expectations are more profound in hard-to-reach areas, and if so, what strategies could be adopted to facilitate trusting relationships in these frequently neglected areas.

### Other factors affecting interpersonal relationships

HSAs operated within a complex network of vertical programmes, which came with different (or sometimes similar) supervisors and volunteers. This led to multiple reporting mechanisms and lack of clarity regarding roles, also reported in other settings [43]. Accountability structures at the community level were not well-established and those from the health sector side were executed irregularly and lacked coordination. Poor accountability structures on their own can hinder HSAs’ relationships with both the community and health sector, something that we were not able to confirm in our study and needs further research. The expansion of facility-based tasks as a result of task-shifting hindered HSAs’ relationships with their communities [9]. Although most HSAs recognized their intermediary position, some reported that they saw volunteers as the intermediaries, while they perceived themselves as government workers who are not necessarily supposed to stay in the village. Such viewpoints could hinder relationships with the community, resulting in further mistrust and disconnection [44]. Strategies to support relationships between CHWs and the community need to be taken into account in the current era, where CHWs are introduced as an official cadre in many African health systems [45, 46].

### Study limitations

This study is limited by several factors. The initial purpose was broad: encompassing all factors that could influence HSA performance. Issues related to relationships were derived from this broader research and thus it is possible that some in-depth questions could have been further discussed and probed. As in all qualitative studies there is a possibility of social desirability bias. We tried to avoid this; the experienced research team carried out in-depth probing and conducted the interviews and FGDs in neutral environments. The outcomes of this study cannot easily be generalized to other, for example, urban settings. The study did not find any differences in perspectives of study participants based on study site or personal characteristics. Our study focused on interpersonal relationships between HSAs, the community and health sector and trust came out as an important underlying factor. However, trust is also influenced by interpersonal relationships among HSAs, personalities and the historical, cultural and socio-political context of the health system [42], factors that we did not research.

## Conclusions

Our findings highlight the critical importance of social relationships and behaviours for health systems, a view supported by other scholars [22]. Social relationships and behaviours are particularly critical for CHWs, as intermediaries between communities and the health sector. HSAs’ intermediary position means that they have multiple relationships to manage and build with implications for their performance. This study identified several programme design elements and processes that could facilitate HSA’s interpersonal relationships with actors in the community and health sector. However, support systems were not functioning optimally, due in part to mistrust between different actors within the health system. Trust has to be actively produced and negotiated. Transparency about the roles and responsibilities of HSAs, selection of HSAs and volunteers for trainings, allowances attached to various programmes and more supportive supervision and functional accountability structures are needed to improve communication and dialogue, increase trust and manage expectations between all levels. In this way, the value of HSAs’ unique intermediary position can be maximized and equitable access to health services in hard-to-reach areas and beyond realized.

### Ethics approval and consent to participate

The study was approved by the Royal Tropical Institute Ethical Review Committee in the Netherlands (S45B) and the National Health Sciences Research Committee in Malawi (NHSRC #1168). All study participants gave informed oral or written consent.

### Availability of data and materials

The data supporting the conclusions of this article are available upon request from the corresponding author.

## Additional file

Additional file 1: Topic guide SSI – Close-to-community (CTC) providers: Health Surveillance Assistants (HSAs). (DOCX 20 kb)

## Abbreviations

CHW: community health worker
FGD: focus group discussion
HSA: health surveillance assistant
iCCM: integrated community case management
NGO: non-governmental organization
VHC: village health committee

## Footnotes

1 Defined as areas more than 8 km from a health facility or difficult to access because of geographical terrain or natural barriers.

2 Each district is divided into traditional authorities, led by traditional leaders. Under these, there are group village headmen and village headmen.

3 Lack of trust can be described as distrust or mistrust. Distrust can be defined as a healthy skepticism, while mistrust comprises a more unhealthy cynicism driven by actual or suspected misdeeds [47].
