# Supplementary material for: Health surveillance assistants as intermediates between the community and health sector in Malawi: exploring how relationships influence performance
Source: BMC Health Serv Res. 2016 May 3;16:164. doi: 10.1186/s12913-016-1402-x (PMC4853867; doi:10.1186/s12913-016-1402-x)
Supplement: Additional file 1: — Topic guide SSI – Close-to-community (CTC) providers: Health Surveillance Assistants (HSAs). (DOCX 20 kb) [file 12913_2016_1402_MOESM1_ESM.docx]

## Topic guide SSI – Close-to-community (CTC) providers: Health Surveillance Assistants (HSAs)

1. Take individual consent
2. Fill in coversheet

**Selection and recruitment**

What made you decide to join the programme?

What attracted you?

What criteria were used for selection for your job/role?

Probe for: role community, health professionals, ministry, NGO/CBO

Looking back what do you think about each of these criteria?

If you had to make the criteria for new providers what changes would you make, if any, in the criteria or process of recruitment? Why?

**Incentives/remuneration/motivation**

What things make you feel good or not so good about your work?

Probe for: the contribution they make through their work; the support or incentives they receive e.g. social status**,** livelihood support, economic benefits, other rewards;

Concerns regarding incentives/ remuneration?

Probe for feelings about a voluntarism and regular remuneration**.**

Would you like your children to do this work? Why, why not?

**Motivation continued**

What things influence job satisfaction and motivation and how?

What motivates or demotivates you?

Probe for:

Equipment and supplies;

Workload;

Working environment;

Communication;

Equipment and transportation;

Safety;

Sexual harassment;

Career perspective;

Supervision;

Community;

Clients;

Colleagues;

Other health workers.

**Tasks**

What things influence how you feel about the tasks you carry out?

Probe for expectations of community, clients, other providers and supervisors; how they feel about meeting these expectations; worries; concerns; what happens if something goes wrong, if a client complaints? If appropriate, probe for legislation of clinically related tasks.

**Clients and community**

Who are your clients?

Please draw a map of the village/ward and indicate where the clients come from.

Explore who lives in each of the areas indicated and the ones where no clients come from.

Explore why.

If other CTC providers/ HSAs cover other parts, ask if any area is left out and why.

Explore who in the community is supportive how, why?

**Facilitators and barriers**

What form barriers to the work?

What do you think goes really well in your work?

Give examples?

What things help to make this work go well?

Give an example of how this works?

What do you think does not always go well?

Can you give an example?

What things are influencing that this work does not go well?

Can you give an example?

When examples are given try to probe for reasons that may influence the work if they do not come spontaneous.

**Lessons learned, opportunities and constraints**

Thinking about your work and what can be done to improve it; what would you suggest?

How could this be done?

If we want to start a programme such as yours in the other district what should be done to make the programme work?

What should be avoided?

**The following are specific issues to be explored if not already addressed:**

**Supervision**

How does supervision take place?

Probe for: What do you like about supervision and what do you dislike about supervision? How often are you supervised?

When was the last time?

What happened?

**Control at work**

How are you enabled and limited in your control at work?

Probing: Influencing decision making; feeling powerless; problem solving process.

**General wellbeing and home-work interface**

What do you feel is influencing your wellbeing?

Probe for closeness to family; relative importance of work and home life; workload (multitasks outside HSAs’ work at home and in the community); ways and importance of relaxation and recreation.

**Quality of care**

What do you think about the quality of care in general of the health services provided?

How is the quality of your work evaluated?

By whom? How?

How do you feel about this?

Do you get feedback?

Probe for guidelines, protocols, monitoring of quality

What do you think people in the community think about the quality of health services?

About your work?

How do you know what the community or clients think about the services you provide?

What do they like best?

What do they complain about?

**Communication and interaction with colleagues**

How is the communication and interactions with colleagues (all cadres including supervisors, in charges, volunteers and traditional birth attendants)

How does this influence job satisfaction and motivation?

Probe for: communication channels; meetings; informal contacts; how often; what is the communication about.

What are your feelings about the communication?

**Referral**

What do you do when a client has a problem you cannot solve?

Who do you refer to?

How does the referral process work?

Probe for: different referral processes for different conditions, ask for examples.

What goes well and not so well in referral?

Why? Examples?

**M&E**

What records for you or others do you keep of your work?

How is this information collected?

What communication channels are used?

What happens with this information?

Do you get feedback about the results of your work?

If so, how is this communicated by whom?

**Mobile health**

Do you use mobile technology (e.g. phones) in for your work?

What do you use it for?

Probe for different use: to collect and send information; to coordinate things; to seek advice from others; to contact clients. For each find out what, with who, how often.

Who bought the device?

Who pays for the costs of use, charging etc.?

How do you feel about the use of these devices: advantages, disadvantages?
